# Extraversion and Agreeableness as Predictors of Digital addiction and Work Performance

**DOI:** 10.1192/j.eurpsy.2026.10828

**Published:** 2026-07-31

**Authors:** N. Rmadi, R. Affes, A. Kchaou, A. Hrairi, M. Hajjaji, K. Jmal Hammami

**Affiliations:** 1 Occupational medicine department, Hedi Chaker university hospital; 2 Family medecine department, Faculty of medecine of Sfax, Sfax, Tunisia

## Abstract

**Introduction:**

Extraversion and agreeableness are often associated with positive professional outcomes, such as teamwork and communication skills. However, in the digital era, these socially oriented traits may predispose residents to problematic smartphone and social media use, potentially undermining work performance.

**Objectives:**

To investigate whether extraversion and agreeableness influence work performance directly and indirectly through vulnerability to smartphone and social media addiction.

**Methods:**

A cross-sectional study was conducted in 2025 among 429 Tunisian medical residents. Personality was assessed using the Big Five Inventory-20 (BFI-20), digital addiction with the Smartphone Addiction Scale–Short Version (SAS-SV) and the Bergen Social Media Addiction Scale (BSMAS), and job performance with the Individual Work Performance Questionnaire (IWPQ). Mediation models tested the indirect effects of extraversion and agreeableness on performance via digital addiction.

**Results:**

Both extraversion and agreeableness were directly associated with higher performance scores (p<0.001). However, mediation analyses revealed significant indirect negative effects through digital addiction. Extraversion reduced performance via smartphone addiction (β=–0.219, p<0.001) and social media addiction (β=–0.302, p<0.001). Similarly, agreeableness indirectly reduced performance through increased vulnerability to digital overuse (β=–0.298, p<0.001).

**Conclusions:**

Extraversion and agreeableness, while beneficial in professional interactions, paradoxically increase susceptibility to digital addiction, which diminishes their positive impact on job performance. Residency programs should promote awareness of these risks and provide tools for balanced digital use among socially oriented individuals.

**Disclosure of Interest:**

None Declared

